# Editorial: Employee life satisfaction: real world consequences

**DOI:** 10.3389/fpsyg.2023.1288877

**Published:** 2023-10-24

**Authors:** Rebecca Merkin

**Affiliations:** Department of Communication Studies, Baruch College—CUNY New York, New York, NY, United States

**Keywords:** life satisfaction, organizational politics, health, burnout, anxiety, sports (attitudes toward)

Life satisfaction is defined as “the personal judgment of one's life and is understood as the cognitive component of subjective well-being” (López-Gómez et al., 2020, p. 275). Employee life satisfaction is a fundamental characteristic of employees that is responsible for positive real-world consequences such as increases in organizational citizenship behaviors, feelings of greater calm and less emotional exhaustion, trusting coworkers, and increases in participation at work (Merkin, 2020). Life satisfaction has also been linked to organizational outcomes of performance (e.g., Fritz et al., 2010; Greguras and Diefendorff, 2010; Erdogan et al., 2012), commitment (Murphy et al., 2006; Yilmaz, 2008; Lambert et al., 2009), and turnover (Shaw and Gupta, 2001). Findings show that high levels of life satisfaction are also related to high levels of job satisfaction, lower work tedium, and more work achievements (Adler and Golan, 1981; Walsh et al., 2023).

On the other hand, if work situations result in employees' dissatisfaction with life, the opposite of the positive outcomes above could lead to lower job satisfaction and fewer work achievements (Walsh et al., 2023) as well as increases in turnover propensity (Erdogan et al., 2012), for example. Likely practical negative outcomes from threats to life satisfaction highlight the need for expanding researchers inquiries into the effects that result from decreases in life satisfaction. Consequently, within this collection we consider realistic factors that influence employee's life satisfaction while providing empirical support for further heuristic studies.

Drory et al. showed how abusive leadership impacts employee life satisfaction to such an extent that employees exposed to it display fewer organizational citizenship behaviors, greater counterproductive work behaviors, as well as greater turnover intensions among other findings. What is more, Drory et al. highlight how important organizational politics is in terms of its influence on employees communication tendencies. Their study points out that besides the direct effects abusive leadership has on employee's actions, organizational politics could also prevent or impede the influence of a workers' immediate supervisor in that politics could change workers impressions and assumptions based on their organizational climate.

Yildiz et al. describe how given the COVID-19 epidemic climate, employees working in health care—in particular nurses—when challenged with an increased workload while also being required to participate in obligatory organization citizenship behaviors experiencing significant damage in terms of their mental and physical health. Needless to say, such conditions significantly threatened health care workers' life satisfaction. Thearetically basing their analysis on Kong and Drew (2016) resource depletion theory, Yildiz et al. findings showed that mandatory citizenship behaviors induced moral detachment which was mediated by employees' anger toward the organization. Thus, negatively charged sentiments (as in the case of anger, fear, and sadness) induced moral disengagement by deteriorating workers' self-control. Results showed that when employees used their energy to control their anti-social emotions (e.g., anger), their energy became depleted which made them more susceptible to moral disengagement.

Khan et al. found a surprising finding relating to how remote work provided for the life satisfaction of employees with social anxiety disorder. According to their study, Khan et al. findings showed that although social anxiety disorder correlated with greater psychological distance, unlike previous studies, this greater psychological distance didn't impact employees' burnout levels. In addition, workplace attachment orientation (but not by psychological safety) moderated a connection between social anxiety disorder and psychological distance (Khan et al.). Consequently, further research needs to be carried out to study how socially anxious people in the workplace face their psychological issues, so that employers can execute actions to better support them.

Findings support the relationship between life satisfaction and social support (Wan et al., 1996; Kong et al., 2012; Azpiazu Izaguirre et al., 2021). In a similar fashion, Gosch et al. studied cooperation between work colleagues (PeerCare) and their leaders (LeaderCare) via an adapted health-oriented leadership questionnaire as well as SelfCare's effects on general health. Gosch et al. results showed PeerCare's positive influences on general health because of the helpful support behavior procedures implimented at work. Their findings did find effects not conforming to expectations in their finding that extant employee health effects are offset when leaders take on health-specific support behaviors to their employees (StaffCare). On the other hand, Gosch et al. results indicate that the health-specific support behavior procedures of different people support one another. More specifically, this study's findings indicate that the two types of support (StaffCare and PeerCare) enhance each other. One final result showed that StaffCare greatly influences LeaderCare. Unexpectedly, SelfCare was most effective in terms of the support process. This contribution also discusses questions still needed to be addressed as well as the implications given specific consequences of different health-specific support behavior measurements.

The final article in this collection carried out by Forcher et al. discusses the professional soccer context. Specifically, they investigated how offensive playing style (i.e., while in ball possession) influences physical and technical match performance throughout the offensive play. They also examine success-related considerations. This research considered whether playing style could affect successful “work” outcomes (whether players win the game). This study's results indicated that when offensive playing styles are used, the physical and technical match performance is affected strategically but this style ultimately has a limited influence on whether a team is successful at winning. Coaches can relate these findings to real life situations such as developing training content to prime players for the extensive demands of a particular opposing team.

In summary, the articles included in this series provide new insight into situations that impact a person's life satisfaction adversely that tend to have real world consequences. We combined a broad array of work contexts related to possible real-life circumstances including organizational politics, moral disengagement, burnout levels, general health, and game success. There is still a great need for further research examining real-life consequences of life satisfaction.

## Author contributions

RM: Conceptualization, Writing—original draft, Writing—review and editing.
